# Asking for trouble and getting what we ask for: African swine fever

**DOI:** 10.3325/cmj.2021.62.534

**Published:** 2021-10

**Authors:** Charles H. Calisher

I wish I had been alive to meet Robert Eustace Montgomery; I only missed him by four years. Born in 1880 in Great Britain and graduating from the veterinary school of the University of Edinburgh in Scotland, he served the colonial government as a veterinary pathologist. While in this position, in 1917 Montgomery published a paper describing his discovery of what he called “a tick-borne gastro-enteritis of sheep and goats” (1), now known as Nairobi sheep disease. He left East Africa in 1918 for South Africa to replace Sir Arnold Theiler (the father of Max Theiler, who was awarded a Nobel Prize for developing a yellow fever vaccine) as Director of Veterinary Research at what is now the Onderstepoort Veterinary Institute – but left again for East Africa when Theiler was re-appointed. Montgomery’s research on a disease in pigs in Kenya was undertaken during the period 1909 to 1918 when he was the pathologist for the East African Protectorate. He died in 1932 while serving as veterinary adviser to the Colonial Office. His remarkable publication in 1921 of a paper on “a form of swine fever” in East Africa (2) described a disease known today as African swine fever (ASF). Under less than optimum, even primitive, laboratory and field conditions, with essentially no standardized procedures or reagents at hand, Montgomery conducted innovative, meticulous, detailed, and exhaustive studies of ASF virus and the disease it causes in pigs. These are two of the best scientific papers I have ever read.

Pigs, or suids (phylum Chordata, class Mammalia, order Artiodactyla, family Suidae, subfamily Suinae, genus *Sus*), have been classified taxonomically as being members of various species: *Sus ahoenobarbus* (Palawan bearded pig), *Sus barbatus* (Bornean bearded pig), *Sus cebifrons* (Visayan warty pig), *Sus celebensis* (Celebes warty pig or Sulawesi warty pig), *Sus domesticus* (domestic pig; sometimes considered a subspecies of *S. scrofa*), *Sus oliveri* (Oliver's warty pig or Mindoro warty pig), *Sus philippensis* (Philippine warty pig), *Sus scrofa* [Linnaeus, 1758] (Wild boar), and *Sus verrucosus* (Javan warty pig) (https://en.wikipedia.org/wiki/Pig).

Pigs, porcids, boars, or whatever you want to call them, are native to the Eurasian and African continents, ranging from Europe to the Pacific islands. At any given moment, there are at least a billion live pigs, which are important economically and for food in many areas.

World-wide, the five largest purchasers of pork last year were China, Japan, Italy, Germany, and Poland and the five largest exporting countries were Spain, U.S.A., Germany, Canada, and Denmark. Sale of pork is a huge market. For example, estimated 2021 pork production in China was 40 500 000 metric tons, in the European Union it was 24 500 000 metric tons, and in the U.S.A. it was 12 800 000 metric tons. Sales to China increased in 2021 because of the impact of current ASF outbreaks on internal production. Sale of exported pork and pork products by the U.S.A. in 2020 was $7.7 billion dollars. “Everything but the squeal” is a term used at hog packing houses to indicate that almost nothing of the animal goes to waste. The term dates from about the 1860s and is still used. A longer phrase is: “From the snout to the tail—everything but the squeal.” [Why any but the hungriest of people would eat feet, snout, entrails, or other parts of pigs is unclear to me, but I do not understand green hair, expansive tattoos, or rap “music” either.]

Unrelated to ASFV, Nipah virus (family *Paramyxoviridae*, genus *Henipavirus*) is a zoonotic agent whose reservoir is the fruit bat (genus *Pteropus*), also known as the flying fox. It was first recognized in 1999 when an outbreak of fatal illness occurred in pigs and humans in Malaysia and Singapore. Nearly 300 human cases with more than 100 deaths were recognized and substantial economic impact followed, as more than a million pigs were killed to control the outbreak. Outbreaks have been recorded in some parts of Asia since then, principally in Bangladesh and India.

During that 1999 outbreak a friend at the US Centers for Disease Control and Prevention informally asked me what I thought would happen if Nipah virus reached and spread in China. I had not thought of that at all. There are now more than 1.4 billion people in China and that country is highly dependent on pork as a protein source. ASFV has already reached China and the pig population there has been heavily affected.

Other than humans, pigs are probably the most populous large mammals on the planet and, other than humans, probably the most invasive. Pigs are omnivores, consuming plants and animals. In the wild, they forage for leaves, roots, fruits, and flowers, in addition to some insects and fish but they will eat just about anything, damaging the environment as they move. Domestic pigs that have escaped from urban areas or were allowed to forage in the wild, and in some cases wild boars that were introduced as prey for hunting, have given rise to large populations of feral pigs in North and South Americas, Australia, New Zealand, Hawaii, and other areas where pigs are not native. “Factory farming” of pigs, chickens and other birds, cattle and other animals leads to monocultures of these vertebrates which are susceptible to pathogens and to essentially complete die-offs.

Pigs are recognized as being infected with many parasites and agents of diseases that can be transmitted to humans. Examples of such zoonoses include trichinosis, pork tapeworms, cysticercosis, and brucellosis, and pigs may serve as hosts of large concentrations of parasitic ascarid worms in their digestive tracts. Some strains of influenzaviruses are endemic in pigs, and pigs may also acquire human influenzaviruses.

As for African swine fever and the virus that causes it (ASFV), it is an acute viral hemorrhagic disease of domestic swine with mortality rates approaching 100%. Devastating ASF outbreaks and continuing epizootics starting in the Caucasus region (Georgia) and now in the Russian Federation, China, and other parts of Southeast Asia (2007 to date) and eastern Europe highlight its significance. Troubling for the Americas, and sadly, ASFV has invaded the Dominican Republic, Haiti, and Cuba. Its movement elsewhere in this hemisphere is likely to occur in just a matter of time. Large amounts of money have been spent to limit the expansion of cases but complete control has not been possible, partially a social and political quandary (3).

ASF strain Georgia-07 and its derivatives are now endemic in extensive regions of Europe and Asia and continue to be “out of Africa,” a situation that poses a grave if not an existential threat to the swine industry worldwide. While the current concern is Georgia-07, other emerging ASFV strains will threaten the world for the indefinite future. Economic analysis indicates that an ASF outbreak in the US would result in approximately $15 to $23 billion USD in losses, assuming the disease is rapidly controlled and the US is able to reenter export markets within two years. ASF’s potential to spread and become endemic in new regions, its rapid and efficient transmission among pigs, and the relative stability of the causative agent ASFV in the environment all provide significant challenges for disease control. Effective and robust methods, including vaccines to be used for immediate response, are needed immediately.

Over the last century, ASF has been reported from five continents and is dreaded by pig industries worldwide. I was unable to find any useful information regarding ASFV or ASV on the World Health Organization’s website but the World Organization for Animal Health website includes some marginally useful information (*https://www.oie.int/en/disease/african-swine-fever/).*

Numerous publications have extolled the virtues of Montgomery’s studies (4) and have reported progress made to control the virus (5) but, to now, the only way to stop this disease is to depopulate all affected or exposed swine herds. Obviously, given the speed of spread of this virus, such methods are insufficient. This is not a satisfactory long-term solution, particularly if the owners of the condemned pigs are impoverished families.

The virus causes a hemorrhagic fever with high mortality rates in domestic pigs; some isolates cause death of infected animals within a week after infection. It persistently infects its natural hosts, warthogs, bushpigs, and soft ticks of the genus *Ornithodoros*, which likely act as a vector, with no disease signs (6). It does not cause disease in humans. ASFV is enzootic in sub-Saharan Africa, occurring in the wild in a cycle of infection between ticks and wild domestic pigs, bushpigs, and warthogs. Interestingly, the disease was first described after European settlers brought pigs into areas enzootic for ASFV, and therefore is an example of an emerging infectious disease.

The causative agent, ASFV, is a large (about 200 nm), double-stranded DNA virus in the order *Asfuvirales*, family *Asfarviridae*, and genus *Asfivirus* and is the only species in the genus. ASFV replicates in the cytoplasm of infected cells. It is the only virus with a double-stranded DNA genome known to be transmitted by arthropods and therefore is considered an arbovirus.

## WHY HAS THERE BEEN NO VACCINE DEVELOPED FOR USE AGAINST THIS DISEASE?

First, and basically, ASFV comprises about 150 genes, many more than most other viruses. That alone is a complex problem. ASFV in blood is stable at room temperature (which differs in laboratories with and without air conditioning, of course) for 140 days and at 21.7 °C for 18 months. ASFV in blood heated to 67.8 °C for three hours remained infectious and feces of an infected animal kept at room temperature for 11 days remained infectious. Ornithodoros ticks, which may live up to 25 years, may remain infected for at least eight years. ASFV remains viable and infectious for at least 10 months in salted, dried meat, hence the need to ask (and trust) immigrants whether they are bringing into a country such items.

Removing one of the many genes of this virus, called 23-NL, results in loss of pathogenicity of the virus and a delay in the appearance of the virus, so may participate in the ability of ASFV to replicate in target cells in the pig, cells in the spleen and lymph nodes. ASFV also has a gene called CD-2, also called T-cell surface antigen, which is a cell adhesion molecule found on the surface of T cells and natural killer cells. The CD-2 protein found in ASFV had never before been found in a virus and may play a role in immunosuppression. [Personal note: I am pleased to be retired; double-stranded DNA viruses are too complicated for me to understand. I am a simple, single-stranded RNA virus person.]

Other genes found in ASFV interfere with apoptosis and possess other immunomodulating properties, buying some time for the virus to replicate (7). The only other animal virus that has genes similar to those in ASFV is Epstein-Barr virus (human herpesvirus 4). Many genes of ASFV can be removed without impacting its replication or transmission, but not CD-2. Multiple strain variations among ASFV complicate the search for proteins that are common among them that might be useful in formulation of a widely protective vaccine. Furthermore, inactivated virus preparations do not protect pigs against infection with ASFV, and live-attenuated vaccines, while stimulating what might seem to be adequate levels of protection, are of questionable safety risks and cause side effects in immunized animals.

ASF is and will remain an enormous problem (8) until a highly innovative Nobel Prize-level solution is found. Until then, it is clear that not enough emphasis and funding has been placed on the control of this devastating disease and we will pay the price for our lack of investment. This will be no surprise.
